# A Quantitative Proteomics Approach to Clinical Research with Non-Traditional Samples

**DOI:** 10.3390/proteomes4040031

**Published:** 2016-10-17

**Authors:** Rígel Licier, Eric Miranda, Horacio Serrano

**Affiliations:** 1 Department of Medicine, San Juan Bautista School of Medicine, Caguas 00727, Puerto Rico; 2 Quantitative Proteomics Laboratory, Comprehensive Cancer Center of Puerto Rico, San Juan 00936, Puerto Rico; eric.miranda@upr.edu; 3 Department of Internal Medicine, University of Puerto Rico, Medical Sciences Campus, San Juan 00936, Puerto Rico

**Keywords:** quantitative proteomics, MS/MS, clinical samples, precision medicine, screening

## Abstract

The proper handling of samples to be analyzed by mass spectrometry (MS) can guarantee excellent results and a greater depth of analysis when working in quantitative proteomics. This is critical when trying to assess non-traditional sources such as ear wax, saliva, vitreous humor, aqueous humor, tears, nipple aspirate fluid, breast milk/colostrum, cervical-vaginal fluid, nasal secretions, bronco-alveolar lavage fluid, and stools. We intend to provide the investigator with relevant aspects of quantitative proteomics and to recognize the most recent clinical research work conducted with atypical samples and analyzed by quantitative proteomics. Having as reference the most recent and different approaches used with non-traditional sources allows us to compare new strategies in the development of novel experimental models. On the other hand, these references help us to contribute significantly to the understanding of the proportions of proteins in different proteomes of clinical interest and may lead to potential advances in the emerging field of precision medicine.

## 1. Introduction

The study of molecules responsible for the mechanisms behind body function, better known as omics, has revolutionized the study of the human body. After the conclusion of the human genome project (2001), efforts have shifted into the study and understanding of the human proteome. The increased sensibility and specificity of the available technology has led to the discovery of an increased number of proteins, making possible the creation of databases for proteomes of different organs as in the case of the Human Eye Proteome Project (HEPP) [1]. The human microbiome project has also been created to understand the correlation between human health and the human microbiome [2]. Increasing our overall knowledge of the proteomic composition of the human body can provide insight into the mechanism involved in pathological states. 

Technological advances in the field of quantitative proteomics have allowed for their use in the study of the mechanisms and treatment of disease. With the new techniques, an increased number and types of biological samples can be analyzed. Each biological sample presents a possible place to discover biomarkers in the study for certain diseases. Table S2 contains a summary of the total identified proteins found in the cited work. Some of these proteins are being considered for their use as biomarkers for disease. Some of the biological samples that can be acquired with noninvasive techniques are cerumen, tears, stools, and saliva. Notably, despite its accessibility, cerumen has not been widely studied as a bio fluid [3]. Other samples such as vitreous humor and aqueous humor are acquired by invasive methods but can provide valuable information into many pathologies, especially, but not limited to, the eye. The biological samples can be used to find new approaches to treatment of disease that can eventually translate into progress in personalized medicine efforts.

This review focuses in the uses of quantitative proteomics methodology in the diagnosis and treatment of disease. It centers on research done in human biological samples to serve as a guide for future research. Although there are a large number of biological samples that are currently being used in research, our discussion is limited to research done with mass spectrometry technology in the following samples: cerumen, saliva, vitreous humor, aqueous humor, tears, nipple aspirate fluid, breast milk, cervicovaginal fluid, nasal secretions, and stools. Table S1 contains a summary of the diseases studied with each sample type in the cited works. 

## 2. Techniques in Quantitative Proteomics

Before addressing the theme of non-conventional body fluids in quantitative proteomics, it is of utmost importance to acknowledge two professionals who revolutionized the study of proteins with their application of mass spectrometry in the field. John B. Fenn and Koichi Tanaka shared a Nobel prize in chemistry (2002) for the development of electrospray ionization (ESI) and matrix-assisted laser desorption/ionization (MALDI), respectively [4]. Their contribution enabled scientist to identify and quantify proteins with a high level of precision. The following part will be dedicated to the understanding of the technology available, giving special consideration to the sample preparation and the mass spectrometry technology for protein analysis (ESI and MALDI). 

Research in proteomics requires profound understanding of each phase of the sample preparation and analysis. Some of the most important steps are sample collection, sample conditioning, mass spectrometry processing, comparison of theoretical data with experimental data, and finally, the use of specialized software that can generate lists and tables with the analyzed data. There are also a number of variables that need to be considered as they can often affect the results. One of them is the denaturing of the sample caused by errors in collection or mishandling. Others include poorly performed digestions, desalting errors, and loss of relevant data due to failure to perform depletions. The mass spectrometry and label method selected for the quantitative proteomics assays can also affect the results. It is for this reason that before developing a study design with this type of technology, it is fundamental to understand what method works best for the desired analysis. This can be achieved by the study of previous research, especially of studies using clinical samples. 

The acquisition of clinical samples is a delicate process as it often involves permits and is limited by its availability. When collecting a sample, it is important to follow previously specified conditions of temperature and storage in order for them not to be contaminated or altered. To achieve a good digestion, it is essential to know the initial concentration of the sample. Reaction salts in each phase must be removed before mass spectrometry analysis so it does not interfere with the sample analysis [5]. One of the uses of biological samples in research is their potential as a source for biomarkers. A biomarker is defined as “any substance, structure or process that can be measured in the body or its products and influence or predict the incidence of outcome or disease.” [6]. In order for a substance to be considered as a potential biomarker, some of the most important qualities it must have are precision, sensibility, and reproducibility [7]. Since it is very difficult to find many of those traits in the molecules studied, there are not many potential biomarkers found despite the apparent availability of proteins in the samples. The increased precision of the technology available can help find new and more abundant quantities of proteins that can eventually become biomarkers. 

As previously mentioned, there are two main technologies available for sample processing in mass spectrometry quantitative proteomics analysis. These are electrospray ionization (ESI) and matrix-assisted laser desorption/ionization (MALDI). Both of these techniques can be used with a variety of mass spectrometers. ESI technology uses electric energy to change ions from a solution into gas phase. Neutral compounds can also be processed with ESI MS by being ionized [8]. After they obtain their charge, the ions travel through the analyzer to the detector which can identify them according to their mass/charge (*m*/*z*) ratio [8]. The signals are then analyzed and recorded as a mass spectrum in a computer [8]. In the case of MALDI MS technology, it uses a laser beam to irradiate a solid sample in an organic matrix. This causes the formation of protonated molecules. The ionized samples then travel through a mass spectrometer that in MALDI technology usually works with time of fight (TOF). This type of analysis eventually translates to the *m*/*z* of the sample [9]. A major setback in the use of mass spectrometry is the high cost associated with acquiring the equipment which limits the type of institutions that can own and use the technology [8].

**Figure 1:**
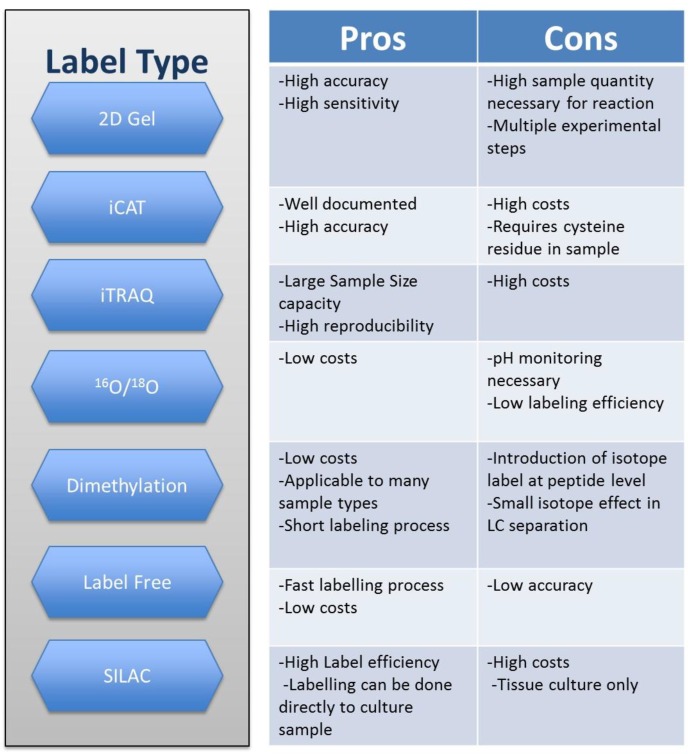
Types of protein labelling for analysis through mass spectrometry and some of their pros and cons [7,12].

An important step in MS quantitative proteomics is the process of sample labelling. Labelling enables the identification and quantification of the samples by different methods. There are two main types of quantification methods: absolute and relative. These are based on the absolute or relative abundance of the samples. Most of the techniques available are part of relative quantification. With stable isotype labelling methods, the quantitative analysis is achieved by calculating the amount of protein with the ratio of peak intensity of isotope ions. The principle behind it is to have samples tagged with stable isotopes so they can be differentiated by their mass. Some of the better known relative quantification isotope strategies are isotope-coded affinity tag (ICAT) isobaric tags for relative and absolute quantification (iTRAQ), dimethyl labeling, ^16^O/^18^O and stable isotope labeling with amino acids in cell culture (SILAC). There is also the label free method in which, as the name suggests, the sample is not labeled [10]. In this technique, the quantity of aprotein is determined by the peak intensity of peptide ions. Even though this type of technique does not require the labelling step and, in theory, can detect more proteins, it lacks precision when compared to the ones mentioned above [10]. Another method that can be used in the quantification of proteins is 2D gel which is widely used in protein separation and quantification [10]. Each method has its advantages and disadvantages that determine the scope of their usage. However, many of the methods have been optimized by their constant usage; eliminating many of the disadvantages initially reported. [7,10,11,12]. Some of the pros and cons of the previously mentioned labelling methods are described in Figure 1. 

At present, the technology for protein identification and quantification is being constantly studied and modified to address the problems encountered in their use. Their use will depend on the availability of the instruments for spectrometry and how well the experimenter knows the labels to be used. It is also important to understand that different variables such as the type of sample and quantity can determine what method is best suited for each experiment. These technologies will hopefully keep advancing and their limitations will continue to be addressed. Other virtues and uses of them will also be discovered as they are used in relatively unexplored samples.

## 3. Ear Wax “Cerumen”

As previously mentioned, the use of cerumen as a biomarker of disease has not been widely studied in the field of quantitative proteomics. In 2013, the first in depth characterization of the proteins present in cerumen in healthy samples was reported [3]. Using three technical approaches, they identified 2013 proteins in human cerumen. For in depth cerumen proteome characterization they used two techniques: peptide prefractionation with online SCX, followed by Liquid chromatography-tandem mass spectrometry (LC-MS/MS), and protein prefractionation with 1D PAGE gel coupled with LC-MS/MS. The in depth characterization of earwax revealed it to be highly complex, comparable to other studied body fluids. It also revealed the presence of proteins that were not previously characterized in mammalian cerumen: serpins, zinc-alpha-2 glycoprotein, apolipoprotein D, and prolactin inducible protein. Mucins were also present in the samples. Most of the proteins previously mentioned have documented immunological functions. The accessibility of cerumen, as well as its rich composition, makes it an adequate sample for the search of biomarkers. However, further investigation is needed in order assess its overall function and biomarker potential [3].

## 4. Saliva

Saliva is one of the most accessible body fluids. It has been studied in the search of biomarkers for a number of diseases, including oral cancer [13,14]. In one study, myosin and actin were evaluated as possible biomarkers for oral cell carcinoma. The study revealed that both proteins showed differential expression in precancerous and cancerous lesions which can help distinguish between them [14]. These findings can eventually help achieve an earlier and targeted treatment that can better the prognosis of oral cancer. 

Immune diseases such as chronic graft vs host disease (CGVH) have also been studied using saliva. CGVH is a complication of allogenic hematopoietic stem cell transplantation that can lead to impaired organ function [6]. It commonly affects the oral cavity and skin. A research study using unstimulated saliva found 102 differently expressed proteins, including downregulation of those associated with oral antimicrobial host immunity. The study used LC-MS/MS labeled free quantification for achieving the results [15]. Alteration of proteins associated with immune response was also found in another study performed with isobaric tags for relative and absolute quantification (iTRAQ) and tandem mass spectrometry (MS/MS) [16]. Additionally, two downregulated proteins were proposed as potential biomarkers: IL-1 receptor antagonist and cystatin B. Another immune related pathology of interest in the search of biomarkers in saliva using quantitative proteomic tools is Sjogren’s syndrome [17,18]. Since there is a lack of clinical tests available for diagnosis, biomarkers found in an accessible body fluid such as saliva would aid in early diagnosis and treatment.

For years, a large amount research into the mechanism and treatment of human immunodeficiency virus (HIV) infection has been reported. Studies based on the quantitative analysis of saliva have been done to search for new ways to monitor the progression of the disease and to understand the mechanism of infection [19]. HIV neurocognitive disorders (HAND) have also been investigated with saliva for the possible involvement of the gut in the pathophysiological mechanisms through quantitative proteomics analysis of the saliva. Using liquid chromatography mass spectrometry, they found 58 proteins that were correlated with cognitive scores. These results reveal apparent oral modulation of brain function during HIV infection [20]. Quantitative proteomic analysis of saliva has also been considered for the development of less invasive glucose monitoring tools in diabetic patients [21] and Down Syndrome comorbidities biomarker discovery [22].

## 5. Vitreous Humor

The human vitreous humor is an aqueous solution that fills the posterior compartment of the eye [23]. Although the substructures of the vitreous and their proteins have been investigated recently [24], studies have centered on using vitreous humor as a diagnostic tool for retinal pathologies. The proximity of the vitreous humor and the retina makes it possible for retinal components to diffuse into the vitreous humor [25] and can make it an ideal setting to find biomarkers for retinal pathologies.

Aside from its many advantages, a major setback to the use of vitreous humor is the invasive techniques for removal of the fluid. This also poses a problem in acquiring samples for study purposes since ethically healthy eyes cannot be subjected to the procedures for the sole purpose of extracting the fluid [1]. To address this issue, researchers have used samples of patients undergoing eye surgery for various diseases but that are presumed to have healthy vitreous humor as controls [25,26] while others have used corneal transplant donated eyes [27,28]. The first study conducted with corneal transplant donated eyes as controls carried out with type 2 diabetes patients [27] revealed 29 differentially expressed proteins: eight of which were increased and 21 which were decreased in proliferative diabetic retinopathy (PDR) patients. With the exclusion of serum proteins, 19 proteins were differentially identified in vitreous fluid of PDR patients compared to those without the disease [27]. Among the proteins identified, the ones that were documented for the first time in vitreous humor were: N(G), N(G)-dimethylarginine dimethylaminohydrolase 1 (DDAH 1), tubulin alpha-1B chain, gamma-enolase, cytosolic acyl coenzyme A thioester hydrolase (ACOT1), malate dehydrogenase (MDH), and phosphatidylethanolamine-binding protein 1 (PEBP1) [27].

The pathways associated with PDR have also been studied with quantitative proteomic techniques [28]. Among their findings, they identified differentially expressed proteins involved in glycolysis/gluconeogenesis, complement and coagulation cascades, gap junction, and phagosome pathways. A research done in 2015 compared proliferative diabetic retinopathy vs non proliferative diabetic retinopathy using free labeled quantitative proteomics analysis [29]. They found 230 proteins that were increased significantly in PDR when compared with non-proliferative retinopathy [29]. In another study [30], 96 proteins from previously published studies were selected to create a list of possible biomarkers for diabetic retinopathy (DR) in PDR and non-proliferative diabetic retinopathy. Based on the results of the study carried out with semi quantitative multiple reaction monitoring (SQ-MRM) and stable isotope dilution with multiple reaction monitoring (SID-MRM), they proposed a protein marker panel composed of APO4, C7, CLU, and ITIH2 for future studies [30]. 

Exudative or wet age related macular degeneration (AMD) is commonly associated with alteration of the retinal pigment epithelium and its basal membrane and can lead to blindness [31]. Using bottom up analysis with capillary electrophoresis–mass spectrometry (CE-MS) and LC-MS/MS for the identification of proteins in the vitreous, a research identified 97 proteins: 19 which were significantly increased in AMD patients [31]. Among the upregulated proteins they found albumin and serotransferrin. Another retinal pathology that has been studied with vitreous humor is idiopathic epiretinal membrane (iEM). To understand the underlying mechanism, reversed phase high-performance liquid chromatography (RP-HPLC) coupled with electrospray ionization tandem mass spectrometry (ESI-MS/MS) were used to analyze vitreous humor samples of patients with (iEM) [32]. 

## 6. Aqueous Humor

As with vitreous humor, aqueous humor (AH) collection cannot be performed in adults that do not suffer from ocular diseases or that are not subjected to eye surgery [1]. Still, it is an excellent source of potential biomarkers for various eye pathologies. One cause of blindness in the elderly population, age related macular degeneration (AMD), has been studied with the use of AH for the possibility of finding biomarkers for early detection. An assessment of the complete composition of the AH of AMD patients was first done in 2012 using Multiple reaction monitoring-mass spectrometry (MRM-MS) [33]. Another research study done using LC-ESI-MS/MS to analyze the AH of AMD patients, reported four proteins that were significantly increased [34]. They proposed Rpn2, one of the proteins found to be over expressed, as a potential biomarker for the disease [34]. A separate study using MALDI-TOF-MS/MS, identified 78 proteins, 68 which were differentially expressed in people with wet AMD vs control [35]. Further research into AH and AMD can yield the desired biomarkers for the disease. In the study of body fluids with quantitative proteomics, protein expression can reveal information regarding the progression of disease. Keratoconus (KC) is a condition that affects the cornea. In KC the cornea adopts a conical shape due to corneal thinning and conical protrusion. In order to better understand the mechanism behind the keratoconus, label free LC-MS/MS quantitative proteomics was used in KC patients and controls [36]. The study found different expression levels in 16 proteins. Some of these proteins were known to play a role in regulation of proteolysis and responses to hypoxia and hydrogen peroxide. Other research done with AH analysis using quantitative proteomics has explored its potential role in the creation of diseases for Juvenile idiopathic arthritis uveitis [37], diabetic retinopathy [38], and branch retinal vein occlusion induced macular edema [39].

## 7. Tears

Tear fluid has been used to study a diverse array of diseases. Unlike vitreous humor and aqueous humor, it is readily accessible and the methods for acquiring it are noninvasive. Dry eyes (DE) is a multifactorial ocular surface disease. The search for understanding more of this pathology has prompted the study of differences in expression of proteins in tear fluid [40,41]. In one study, protein expression in DE was analyzed with iTRAQ and LC-MS/MS and a total of 386 were found [41]. Participants were divided into four groups: non DE (NDE) or control, mild DE (MDE), moderate-to-severe DE (MSDE), and mixed DE (MXDE) [41]. Downregulation of lipocalin-1 lysozyme and prolactin-inducible protein was present in all subgroups of (DE). It was also found that there was an increased amount of downregulated proteins in MSDE when compared with MDE [41]. 

Another approach to the study of DE was done by focusing on the different clinical phenotypes of patients with DE and how they could affect the tear proteome [42]. This research was done using laser desorption/ionization time of flight mass spectrometry (MALDI-TOF/TOF MS). Participants were subdivided into four groups: healthy controls, aqueous-deficient dry eye (DRYaq), lipid-deficient dry eye (DRYlip), and a combination of the two (DRYaqlip). Downregulation of PRR4 and upregulation of mammaglobulin B and lipophilin A was seen in DRYaq patients and DRYaqlip when compared with that of controls and DRYlip patients. There results demonstrated that different clinical phenotypes are associated with different alterations of the tear film proteome [42]. Other research has been done to understand DE associated factors, such as contact lens related dry eye [43]. 

The association of dry eye syndrome with type 2 diabetes was studied using Two-Dimensional Strong Cation-Exchange/Reversed-Phase Nano-Scale LC MS [44]. They found increased expression of proteins in patients with diabetes and dry eye syndrome. Among the proteins with increased expression they found annexin A1, elastase 2, clusterin, and apolipoprotein AII. Diabetic retinopathy, another well-known diabetes complication, has also been studied with tears fluid using a quantitative approach [45]. Recently, a study addressed the search for new biomarkers for the differentiation of thyroid-associated orbithopathy (TAO) and dry eye syndrome [46]. With the use of matrix-assisted laser desorption ionization mass spectrometry, they identified deregulated proteins in TAO and dry eye. Among the findings, downregulated proteins in TAO compared to that of dry eye were reported including proline-rich protein 1, uridine diphosphate glucosedehydrogenase, calgranulin A transcriptionactivator BRG1, annexin A1, cystatin, heat shock protein 27, and galectin [46]. Other diseases than have been studied to define their tear proteome expression differences are primary open angle glaucoma and pseudoexfoliative glaucoma [47].

As previously mentioned, tears have been used to study a variety of disease, including some that are not ocular in nature. The search for non-invasive biomarkers for Alzheimer’s disease has led quantitative proteomics research in tear fluid. Using LC-MS/MS and Selected Reaction Monitoring (SRM) based targeted proteomics, they found that a combination of lipocalin-1, dermicin, lysozyme C, and lacritin as biomarkers showed 81% sensitivity and 77% specificity [48]. Quantitative approaches have also been used to test ocular responses to laser platforms for refractive surgery as they manifest in tear proteins. This type of research can help understand the effect of different surgical procedures on the body [49]. Other pathologies have been studied using tears and quantitative proteomics tools are vernal keratoconjunctivitis [49], multiple sclerosis [50], and primary open glaucoma [51]. 

## 8. Nipple Aspirate Fluid 

Nipple aspirate fluid (NAF) is a ductal fluid that can be extracted from the breast through the nipple with a non-invasive technique [52]. NAF has primarily been studied as a source for cancer biomarkers. The need for better diagnostic tools for early breast cancer detection coupled with the non-invasiveness of the technique of NAF extraction makes it a promising source of biomarkers. However, each sample yields a low abundance of proteins which affects its processing. This setback has been addressed by studying different pre-fractionation technique platforms for NAF, providing methodological information for future quantitative studies [53].

In the early 2000s, several studies in the field of proteomics explored the use of NAF as a source of breast cancer biomarkers [54,55,56]. More recently, quantitative liquid chromatography tandem mass spectrometry (LC-MS/MS) was used to compare the parent estrogen and their metabolites in nipple aspirate fluid, ductal lavage supernatant, and serum in BRCA1/2 mutation carriers [57]. The goal of this study was to compare measurements of estradiol and estrone metabolites EM and parent estrogens (PE) in different samples for biomarker discovery to see which sample provided better results. Although serum yielded the most promising results, further studies comparing biological samples could generate more comprehensive data of the biomarkers discovered in all of them. 

## 9. Breast Milk/Colostrum

Breast milk as a source of biomarkers for disease has been relatively unexplored in the field of quantitative proteomics. Several research studies have assessed the contents of the fluid [58,59,60,61]. A study using liquid chromatography tandem mass spectrometry investigated how gestational diabetes mellitus (GEM) affects breast milk components. Through analysis of colostral whey, they identified 27 proteins of interests, 10 of which were differentially expressed in GEM patients [62]. 

## 10. Cervicovaginal Fluid

Cervicovaginal fluid (CVF) originates from the vagina, cervix, endometrium, and oviducts [63]. Over the years, several research projects have focused on identifying the proteomic composition of CVF [63,64]. CVF has been known to play a crucial role in the innate immune defense as seen with studies regarding HIV transmission [65]. An iTRAQ based study was conducted to analyze CFV from HIV-exposed seronegative individuals (HESN) that were at high risk compared to low risk (HESN) and HIV positive patients. The research revealed that Serpin A5 was up regulated and Myeloblastin was downregulated in high risk HESN when compared to that in the other groups [66]. Further study into the mechanism behind HIV resistance and development of treatments is recommended [66]. 

CVF has also been studied for its potential use in cervical cancer screening. Using label free quantitative analysis as well as qualitative identification a study found alpha-actinin-4 as a potential biomarker in CVF for cervical cancer [67]. CVF is easily accessible and has a number of potential biomarkers that makes it ideal for the development of self-diagnostic testing for cervical cancer. 

## 11. Nasal Secretions

The protein content of nasal secretions has been analyzed in a number of research studies [68,69]. A chronic rhinosinusitis (CRS) study with pediatric patients found a total of 294 proteins [70]. As they hypothesized, the researchers found an increased expression of MUC5B in CRS patients when compared to that of controls. The study was carried out with high performance liquid chromatography and linear ion trap mass spectrometer (HPLC-LIT-MS) and validated with Western blot analysis [70].

## 12. Broncho Alveolar Lavage Fluid 

Broncho alveolar lavage fluid (BALF) has been used in the study of pulmonary diseases. Idiopathic pulmonary fibrosis (IPF) is a chronic, progressive interstitial pneumonia with poor prognosis. To understand more about the underlying mechanism of the pathology and differentiate between other fibrocystic pneumonia candidate, biomarkers in BALF have been investigated [71]. The first gel free quantitative analysis of BALF in IPF patients found upregulation of probiotic cytokine CCL24 and an overexpression of osteopontine [72]. 

Chronic obstructive pulmonary disease (COPD) is a complex and heterogeneous disease with major public health importance. A study using nano-reverse phase liquid chromatography mass spectrometer (RPLC/MS) found 423 proteins, 76 of which displayed altered expression. They also observe upregulated expression of alcohol metabolism enzymes, including ADH1B, ALDH2, and ALDH3A1. This was the first report of the association between alcohol metabolism and COPD and its possible implications [73]. Another study focused on the association between lung cancer and COPD, using matrix-assisted laser desorption/ionization-time of flight (MALDI:TOF/TOF) to help with the understanding of the pathogenic pathways of both diseases and possible protein biomarkers [74]. 

The use of BALF has also been explored to help with the understanding of some inflammatory and immune diseases. Acute respiratory distress syndrome (ARDS) is caused by a response to infection or other inflammatory triggers and has a high mortality rate. Currently, there are no biomarkers for ARDS that can provide prognostic information for clinical management. To investigate new possible biomarkers, a study used iTRAQ and LC-MS/MS to compare BALF protein content in different stages from the disease [75]. Their findings demonstrated differences in absolute protein levels in the different stages studied [75]. 

Chronic graft dysfunction is a complication of lung transplantation and is the major cause of morbidity and mortality in transplant patients. The search for non-invasive biomarkers has led to the investigation of BALF with the use of quantitative proteomic techniques. Lung cancer is another area of interest for BALF research. Primary lung cancer adenocarcinoma has a poor prognosis. A study using liquid chromatography mass spectrometry found 33 overexpressed proteins in samples taken from patients that are potential biomarkers in the future [76]. 

## 13. Stools

The search for biomarkers for different diseases that affect the gastrointestinal (GI) tract has led to the study and characterization of proteins from human stools. There is a special interest in the host-microbe interaction as it can provide insight into pathophysiological mechanisms of GI disease [77,78]. There are several investigations in the infant gut microbiome [79,80]. The complexity of the processes occurring in stool have inspired interdisciplinary research techniques for their understanding. This was the case in a study addressing the relationship between gut microbes in obese children and fatty liver disease [81]. One of the techniques used in this project was nano LC-2D coupled to thermo orbitrap XL tandem mass spectrometry, mainly used to understand the metabolic and proteomic data from microbial samples [81]. A similar approach was undertaken to study the microbiome role in the development of Crohn’s disease [82]. One of the setbacks in stool samples is the presence of human and microbe proteins in the sample. In one study done to characterize the human infant microbiome, they addressed this problem by proposing a new strategy to enhance the microbial characterization [79]. 

## 14. Conclusions

Quantitative proteomics has provided the tools necessary to explore the mechanisms of the human body and their changes in disease. Biological samples can lead to the creation of better and less invasive screening methods. Still, the quantitative proteomic analysis of the samples previously discussed is constantly evolving, with new technologies and approaches to address the problems that are encountered in their use. In the search for future analysis methods it will be important to first understand how the technology could be used for studying the mechanisms of disease and discovering biomarkers for their identification. In this manner, effort will be aimed at specific goals that will help us achieve faster and better results.

## Supplementary Materials

The following are available online at www.mdpi.com/2227-7382/4/4/31/s1.

## References

[1] Semba R.D., Enghild J.J., Venkatraman V., Dyrlund T.F., Eyk J.E. (2013). The human eye proteome project: Perspectives on an emerging proteome. Proteomics.

[2] Bennike T., Birkelund S., Stensballe A., Andersen V. (2014). Biomarkers in inflammatory bowel diseases: Current status and proteomics identification strategies. World J. Gastroenterol.

[3] Feig M.A., Hammer E., Volker U., Jehmlich N. (2013). In-depth proteomic analysis of the human cerumen-a potential novel diagnostically relevant biofluid. J. Proteom..

[4] Vestling M.M. (2003). Using mass spectrometry for proteins. J. Chem. Educ..

[5] Bonzon-Kulichenko E., Pérez-Hernández D., Núñez E., Martínez-Acedo P., Navarro P., Trevisan-Herraz M., del Carmen Ramos M., Sierra S., Martínez-Martínez S., Ruiz-Meana M. (2011). A robust method for quantitative high-throughput analysis of proteomes by 18O labeling. Mol. Cell. Proteom..

[6] World Health Organization (2001). Biomarkers in risk assessment: Validity and validation. Environmental Health Criteria.

[7] Chahrour O., Cobice D., Malone J. (2015). Stable isotope labelling methods in mass spectrometry-based quantitative proteomics. J. Pharm. Biomed. Anal..

[8] Ho C., Lam C., Chan M., Cheung R., Law L., Lit L., Ng K., Suen M., Tai H. (2003). Electrospray ionisation mass spectrometry: Principles and clinical applications. Clinic. Biochem. Rev..

[9] Andersson M., Andren P., Caprioli R., Alzate O. (2010). Maldi imaging and profiling mass spectrometry in neuroproteomics. Neuroproteomics.

[10] Megger D.A., Bracht T., Meyer H.E., Sitek B. (2013). Label-free quantification in clinical proteomics. Biochim. Biophys. Acta (BBA)-Proteins Proteom..

[11] Kito K., Ito T. (2008). Mass spectrometry-based approaches toward absolute quantitative proteomics. Curr. Genom..

[12] Boersema P.J., Raijmakers R., Lemeer S., Mohammed S., Heck A.J. (2009). Multiplex peptide stable isotope dimethyl labeling for quantitative proteomics. Nat. Protoc..

[13] Hu S., Yu T., Xie Y., Yang Y., Li Y., Zhou X., Tsung S., Loo R.R., Loo J.A., Wong D.T. (2007). Discovery of oral fluid biomarkers for human oral cancer by mass spectrometry. Cancer Genom.-Proteom..

[14] De Jong E.P., Xie H., Onsongo G., Stone M.D., Chen X.B., Kooren J.A., Refsland E.W., Griffin R.J., Ondrey F.G., Wu B. (2010). Quantitative proteomics reveals myosin and actin as promising saliva biomarkers for distinguishing pre-malignant and malignant oral lesions. PLoS ONE.

[15] Bassim C.W., Ambatipudi K.S., Mays J.W., Edwards D.A., Swatkoski S., Fassil H., Baird K., Gucek M., Melvin J.E., Pavletic S.Z. (2012). Quantitative salivary proteomic differences in oral chronic graft-versus-host disease. J. Clin. Immunol..

[16] Devic I., Shi M., Schubert M.M., Lloid M., Izutsu K.T., Pan C., Missaghi M., Morton T.H., Mancl L.A., Zhang J. (2014). Proteomic analysis of saliva from patients with oral chronic graft-versus-host disease. Biol. Blood Marrow Transplant..

[17] Ambatipudi K.S., Swatkoski S., Moresco J.J., Tu P.G., Coca A., Anolik J.H., Gucek M., Sanz I., Yates J.R., Melvin J.E. (2012). Quantitative proteomics of parotid saliva in primary sjögren’s syndrome. Proteomics.

[18] Hu S., Wang J., Meijer J., Ieong S., Xie Y., Yu T., Zhou H., Henry S., Vissink A., Pijpe J. (2007). Salivary proteomic and genomic biomarkers for primary sjögren’s syndrome. Arthr. Rheum..

[19] Zhang N., Zhang Z., Feng S., Wang Q., Malamud D., Deng H. (2013). Quantitative analysis of differentially expressed saliva proteins in human immunodeficiency virus type 1 (HIV-1) infected individuals. Anal. Chim. Acta.

[20] Dominy S.S., Brown J.N., Ryder M.I., Gritsenko M., Jacobs J.M., Smith R.D. (2014). Proteomic analysis of saliva in hiv-positive heroin addicts reveals proteins correlated with cognition. PLoS ONE.

[21] Bencharit S., Baxter S.S., Carlson J., Byrd W.C., Mayo M.V., Border M.B., Kohltfarber H., Urrutia E., Howard-Williams E.L., Offenbacher S. (2013). Salivary proteins associated with hyperglycemia in diabetes: A proteomic analysis. Mol. BioSyst..

[22] Cabras T., Pisano E., Montaldo C., Giuca M.R., Iavarone F., Zampino G., Castagnola M., Messana I. (2013). Significant modifications of the salivary proteome potentially associated with complications of down syndrome revealed by top-down proteomics. Mol. Cell. Proteom..

[23] Aretz S., Krohne T.U., Kammerer K., Warnken U., Hotz-Wagenblatt A., Bergmann M., Stanzel B.V., Kempf T., Holz F.G., Schnölzer M. (2013). In-depth mass spectrometric mapping of the human vitreous proteome. Proteom. Sci..

[24] Skeie J.M., Roybal C.N., Mahajan V.B. (2015). Proteomic insight into the molecular function of the vitreous. PLoS ONE.

[25] Gao B.-B., Chen X., Timothy N., Aiello L.P., Feener E.P. (2008). Characterization of the vitreous proteome in diabetes without diabetic retinopathy and diabetes with proliferative diabetic retinopathy. J. Proteom. Res..

[26] Kim K., Kim S.J., Yu H.G., Yu J., Park K.S., Jang I.J., Kim Y. (2009). Verification of biomarkers for diabetic retinopathy by multiple reaction monitoring. J. Proteom. Res..

[27] Wang H., Feng L., Hu J.W., Xie C.L., Wang F. (2012). Characterisation of the vitreous proteome in proliferative diabetic retinopathy. Proteom. Sci..

[28] Wang H., Feng L., Hu J., Xie C., Wang F. (2013). Differentiating vitreous proteomes in proliferative diabetic retinopathy using high-performance liquid chromatography coupled to tandem mass spectrometry. Exp. Eye Res..

[29] Loukovaara S., Nurkkala H., Tamene F., Gucciardo E., Liu X., Repo P., Lehti K., Varjosalo M. (2015). Quantitative proteomics analysis of vitreous humor from diabetic retinopathy patients. J. Proteom. Res..

[30] Jin J., Min H., Kim S.J., Oh S., Kim K., Yu H.G., Park T., Kim Y. (2016). Development of diagnostic biomarkers for detecting diabetic retinopathy at early stages using quantitative proteomics. J. Diabetes Rev..

[31] Koss M.J., Hoffmann J., Nguyen N., Pfister M., Mischak H., Mullen W., Husi H., Rejdak R., Koch F., Jankowski J. (2014). Proteomics of vitreous humor of patients with exudative age-related macular degeneration. PLoS ONE.

[32] Yu J., Feng L., Wu Y., Wang H., Ba J., Zhu W., Xie C. (2014). Vitreous proteomic analysis of idiopathic epiretinal membranes. Mol. BioSyst..

[33] Kim T.W., Kang J.W., Ahn J., Lee E.K., Cho K.-C., Han B.N.R., Hong N.Y., Park J., Kim K.P. (2012). Proteomic analysis of the aqueous humor in age-related macular degeneration (amd) patients. J. Proteom. Res..

[34] Lee H., Choi A.J., Kang G.-Y., Park H.S., Kim H.C., Lim H.J., Chung H. (2014). Increased 26s proteasome non-atpase regulatory subunit 1 in the aqueous humor of patients with age-related macular degeneration. BMB Rep..

[35] Yao J., Liu X., Yang Q., Zhuang M., Wang F., Chen X., Hang H., Zhang W., Liu Q. (2013). Proteomic analysis of the aqueous humor in patients with wet age-related macular degeneration. Proroteom.-Chin Appl..

[36] Soria J., Villarrubia A., Merayo-Lloves J., Elortza F., Azkargorta M., de Toledo J.A., Rodriguez-Agirretxe I., Suarez T., Acera A. (2015). Label-free LC–MS/MS quantitative analysis of aqueous humor from keratoconic and normal eyes. Mol. Vis..

[37] Ayuso V.K., de Boer J.H., Byers H.L., Coulton G.R., Dekkers J., de Visser L., van Loon A.M., Schellekens P.A., Rothova A., de Groot-Mijnes J.D. (2013). Intraocular biomarker identification in uveitis associated with juvenile idiopathic arthritisjia-associated uveitis biomarker identification. Investig. Ophthalmol. Vis. Sci..

[38] Chiang S.-Y., Tsai M.-L., Wang C.-Y., Chen A., Chou Y.-C., Hsia C.-W., Wu Y.-F., Chen H.-M., Huang T.-H., Chen P.-H. (2012). Proteomic analysis and identification of aqueous humor proteins with a pathophysiological role in diabetic retinopathy. J. Proteom..

[39] Yao J., Chen Z., Yang Q., Liu X., Chen X., Zhuang M., Liu Q. (2013). Proteomic analysis of aqueous humor from patients with branch retinal vein occlusion-induced macular edema. Int. J. Mol. Med..

[40] Zhou L., Beuerman R.W., Chan C.M., Zhao S.Z., Li X.R., Yang H., Tong L., Liu S., Stern M.E., Tan D. (2009). Identification of tear fluid biomarkers in dry eye syndrome using itraq quantitative proteomics. J. Proteom. Res..

[41] Srinivasan S., Thangavelu M., Zhang L., Green K.B., Nichols K.K. (2012). Itraq quantitative proteomics in the analysis of tears in dry eye patientsanalysis of tears in dry eye patients. Investig. Ophthalmol. Vis. Sci..

[42] Boehm N., Funke S., Wiegand M., Wehrwein N., Pfeiffer N., Grus F.H. (2013). Alterations in the tear proteome of dry eye patients—a matter of the clinical phenotypetear proteome of dry eye patients. Investig. Ophthalmol. Vis. Sci..

[43] Nichols J.J., Green-Church K.B. (2009). Mass spectrometry-based proteomic analyses in contact lens-related dry eye. Cornea.

[44] Li B., Sheng M., Xie L., Liu F., Yan G., Wang W., Lin A., Zhao F., Chen Y. (2014). Tear proteomic analysis of patients with type 2 diabetes and dry eye syndrome by two-dimensional nano-liquid chromatography coupled with tandem mass spectrometrynano-liquid chromatography/tandem mass spectrometry. Investig. Ophthalmol. Vis. Sci..

[45] Csősz É., Boross P., Csutak A., Berta A., Tóth F., Póliska S., Török Z., Tőzsér J. (2012). Quantitative analysis of proteins in the tear fluid of patients with diabetic retinopathy. J. Proteom..

[46] Matheis N., Grus F.H., Breitenfeld M., Knych I., Funke S., Pitz S., Ponto K.A., Pfeiffer N., Kahaly G.J. (2015). Proteomics differentiate between thyroid-associated orbitopathy and dry eye syndromeproteomics of tears. Investig. Ophthalmol. Vis. Sci..

[47] Pieragostino D., Bucci S., Agnifili L., Fasanella V., D'Aguanno S., Mastropasqua A., Ciancaglini M., Mastropasqua L., di Ilio C., Sacchetta P. (2012). Differential protein expression in tears of patients with primary open angle and pseudoexfoliative glaucoma. Mol. BioSyst..

[48] Kalló G., Emri M., Varga Z., Ujhelyi B., Tőzsér J., Csutak A., Csősz É. (2016). Changes in the chemical barrier composition of tears in alzheimer’s disease reveal potential tear diagnostic biomarkers. PLoS ONE.

[49] D’Souza S., Petznick A., Tong L., Hall R.C., Rosman M., Chan C., Koh S.K., Beuerman R.W., Zhou L., Mehta J.S. (2014). Comparative analysis of two femtosecond lasik platforms using itraq quantitative proteomicstear protein profile in lasik. Investig. Ophthalmol. Vis. Sci..

[50] Salvisberg C., Tajouri N., Hainard A., Burkhard P.R., Lalive P.H., Turck N. (2014). Exploring the human tear fluid: Discovery of new biomarkers in multiple sclerosis. Proteom.-Chin Appl..

[51] Pieragostino D., Agnifili L., Fasanella V., D'Aguanno S., Mastropasqua R., Di Ilio C., Sacchetta P., Urbani A., Del Boccio P. (2013). Shotgun proteomics reveals specific modulated protein patterns in tears of patients with primary open angle glaucoma naive to therapy. Mol. BioSyst..

[52] Zangar R.C., Varnum S.M., Covington C.Y., Smith R.D. (2004). A rational approach for discovering and validating cancer markers in very small samples using mass spectrometry and elisa microarrays. Dis. Markers.

[53] Brunoro G.V.F., Carvalho P.C., da Silva Ferreira A.T., Perales J., Valente R.H., de Moura Gallo C.V., Pagnoncelli D., da Costa Neves-Ferreira A.G. (2015). Proteomic profiling of nipple aspirate fluid (NAF): Exploring the complementarity of different peptide fractionation strategies. J. Proteom..

[54] Paweletz C.P., Trock B., Pennanen M., Tsangaris T., Magnant C., Liotta L.A., Petricoin III E.F. (2001). Proteomic patterns of nipple aspirate fluids obtained by seldi-tof: Potential for new biomarkers to aid in the diagnosis of breast cancer. Dis. Markers.

[55] Alexander H., Stegner A.L., Wagner-Mann C., du Bois G.C., Alexander S., Sauter E.R. (2004). Proteomic analysis to identify breast cancer biomarkers in nipple aspirate fluid. Chin. Cancer Res..

[56] Sauter E.R., Shan S., Hewett J.E., Speckman P., du Bois G.C. (2005). Proteomic analysis of nipple aspirate fluid using seldi-tof-ms. Int. J. Cancer.

[57] Loud J.T., Gierach G.L., Veenstra T.D., Falk R.T., Nichols K., Guttmann A., Xu X., Greene M.H., Gail M.H. (2014). Circulating estrogens and estrogens within the breast among postmenopausal *BRCA_1/2_* mutation carriers. Breast Cancer Res. Treat..

[58] Coscia A., Orrù S., Di Nicola P., Giuliani F., Rovelli I., Peila C., Martano C., Chiale F., Bertino E. (2012). Cow’s milk proteins in human milk. J. Biol. Regul. Homeost. Agents.

[59] Coscia A., Orrù S., Di Nicola P., Giuliani F., Varalda A., Peila C., Fabris C., Conti A., Bertino E. (2012). Detection of cow’s milk proteins and minor components in human milk using proteomics techniques. J. Matern.-Fetal Neonatal Med..

[60] Liao Y., Alvarado R., Phinney B., Lonnerdal B. (2011). Proteomic characterization of human milk whey proteins during a twelve-month lactation period. J. Proteom. Res..

[61] Liao Y., Alvarado R., Phinney B., Lonnerdal B. (2011). Proteomic characterization of specific minor proteins in the human milk casein fraction. J. Proteom. Res..

[62] Grapov D., Lemay D.G., Weber D., Phinney B.S., Azulay Chertok I.R., Gho D.S., German J.B., Smilowitz J.T. (2014). The human colostrum whey proteome is altered in gestational diabetes mellitus. J. Proteom. Res..

[63] Shaw J.L., Smith C.R., Diamandis E.P. (2007). Proteomic analysis of human cervico-vaginal fluid. J. Proteom. Res..

[64] Di Quinzio M.K., Oliva K., Holdsworth S.J., Ayhan M., Walker S.P., Rice G.E., Georgiou H.M., Permezel M. (2007). Proteomic analysis and characterisation of human cervico-vaginal fluid proteins. Aust. N. Z. J. Obstet. Gynaecol..

[65] Venkataraman N., Cole A.L., Svoboda P., Pohl J., Cole A.M. (2005). Cationic polypeptides are required for anti-hiv-1 activity of human vaginal fluid. J. Immunol..

[66] Van Raemdonck G., Zegels G., Coen E., Vuylsteke B., Jennes W., Van Ostade X. (2014). Increased serpin A5 levels in the cervicovaginal fluid of HIV-1 exposed seronegatives suggest that a subtle balance between serine proteases and their inhibitors may determine susceptibility to HIV-1 infection. Virology.

[67] Van Raemdonck G.A., Tjalma W.A., Coen E.P., Depuydt C.E., Van Ostade X.W. (2014). Identification of protein biomarkers for cervical cancer using human cervicovaginal fluid. PLoS ONE.

[68] Debat H., Eloit C., Blon F., Sarazin B., Henry C., Huet J.-C., Trotier D., Pernollet J.-C. (2007). Identification of human olfactory cleft mucus proteins using proteomic analysis. J. Proteom. Res..

[69] Casado B., Pannell L.K., Iadarola P., Baraniuk J.N. (2005). Identification of human nasal mucous proteins using proteomics. Proteomics.

[70] Saieg A., Brown K.J., Pena M.T., Rose M.C., Preciado D. (2014). Proteomic analysis of pediatric sinonasal secretions shows increased muc5b mucin in crs. Pediatr. Res..

[71] Hara A., Sakamoto N., Ishimatsu Y., Kakugawa T., Nakashima S., Hara S., Adachi M., Fujita H., Mukae H., Kohno S. (2012). S100a9 in balf is a candidate biomarker of idiopathic pulmonary fibrosis. Respir. Med..

[72] Foster M.W., Morrison L.D., Todd J.L., Snyder L.D., Thompson J.W., Soderblom E.J., Plonk K., Weinhold K.J., Townsend R., Minnich A. (2015). Quantitative proteomics of bronchoalveolar lavage fluid in idiopathic pulmonary fibrosis. J. Proteom. Res..

[73] Tu C., Mammen M.J., Li J., Shen X., Jiang X., Hu Q., Wang J., Sethi S., Qu J. (2013). Large-scale, ion-current-based proteomics investigation of bronchoalveolar lavage fluid in chronic obstructive pulmonary disease patients. J. Proteom. Res..

[74] Pastor M., Nogal A., Molina-Pinelo S., Melendez R., Salinas A., De la Pena M.G., Martin-Juan J., Corral J., Garcia-Carbonero R., Carnero A. (2013). Identification of proteomic signatures associated with lung cancer and copd. J. Proteom..

[75] Bhargava M., Becker T.L., Viken K.J., Jagtap P.D., Dey S., Steinbach M.S., Wu B., Kumar V., Bitterman P.B., Ingbar D.H. (2014). Proteomic profiles in acute respiratory distress syndrome differentiates survivors from non-survivors. PLoS ONE.

[76] Almatroodi S.A., McDonald C.F., Collins A.L., Darby I.A., Pouniotis D.S. (2015). Quantitative proteomics of bronchoalveolar lavage fluid in lung adenocarcinoma. Cancer Genom.-Proteom..

[77] Verberkmoes N.C., Russell A.L., Shah M., Godzik A., Rosenquist M., Halfvarson J., Lefsrud M.G., Apajalahti J., Tysk C., Hettich R.L. (2009). Shotgun metaproteomics of the human distal gut microbiota. ISME J..

[78] Kolmeder C.A., De Been M., Nikkilä J., Ritamo I., Mättö J., Valmu L., Salojärvi J., Palva A., Salonen A., de Vos W.M. (2012). Comparative metaproteomics and diversity analysis of human intestinal microbiota testifies for its temporal stability and expression of core functions. PLoS ONE.

[79] Xiong W., Giannone R.J., Morowitz M.J., Banfield J.F., Hettich R.L. (2014). Development of an enhanced metaproteomic approach for deepening the microbiome characterization of the human infant gut. J. Proteom. Res..

[80] Klaassens E.S., de Vos W.M., Vaughan E.E. (2007). Metaproteomics approach to study the functionality of the microbiota in the human infant gastrointestinal tract. Appl. Environ. Microbiol..

[81] Michail S., Lin M., Frey M.R., Fanter R., Paliy O., Hilbush B., Reo N.V. (2015). Altered gut microbial energy and metabolism in children with non-alcoholic fatty liver disease. FEMS Microbiol. Ecol..

[82] Erickson A.R., Cantarel B.L., Lamendella R., Darzi Y., Mongodin E.F., Pan C., Shah M., Halfvarson J., Tysk C., Henrissat B. (2012). Integrated metagenomics/metaproteomics reveals human host-microbiota signatures of crohn’s disease. PLoS ONE.

