# Supplementary material for: A Quantitative Proteomics Approach to Clinical Research with Non-Traditional Samples
Source: Proteomes. 2016 Oct 17;4(4):31. doi: 10.3390/proteomes4040031 (PMC5260964; doi:10.3390/proteomes4040031)
Supplement: Supplementary file 1 [file proteomes-04-00031-s001.pdf]

A Quantitative Proteomics Approach to Clinical Research with Non-Traditional Samples

Rígel Licier, Eric Mirando and Horacio Serrano

Table S1. Overview of articles cited by sample used.

Sample	Pathology	Year	Reference
Saliva	Oral Cancer	2007	[13]
Saliva	Oral cancer	2010	[14]
Saliva	Chronic Graft vs. Host Disease	2012	[15]
Saliva	Chronic Graft vs. Host Disease	2014	[16]
Saliva	Primary Shrogen Syndrome	2012	[17]
Saliva	Primary Shrogen Syndrome	2007	[18]
Saliva	HIV	2013	[19]
Saliva	HIV	2014	[20]
Saliva	Diabetes	2013	[21]
Saliva	Down Syndrome	2013	[22]
Vitreous Humor	Diabetic Retinopathy	2008	[25]
Vitreous Humor	Diabetic Retinopathy	2009	[26]
Vitreous Humor	Diabetic Retinopathy	2012	[27]
Vitreous Humor	Diabetic Retinopathy	2013	[28]
Vitreous Humor	Diabetic Retinopathy	2015	[29]
Vitreous Humor	Diabetic Retinopathy	2015	[30]
Vitreous Humor	Age-Related Macular Degeneration	2014	[31]
Vitreous Humor	Idiopathic epiretinal Membrane	2014	[32]
Aqueous Humor	Age-Related Macular Degeneration	2012	[33]
Aqueous Humor	Age-Related Macular Degeneration	2014	[34]
Aqueous Humor	Wet Age-Related Macular Degeneration	2013	[35]
Aqueous Humor	Keratoconus	2015	[36]
Aqueous Humor	Juvenile idiopathic arthritis related uveitis	2013	[37]
Aqueous Humor	Diabetic Retinopathy	2012	[38]
Aqueous Humor	Retinal Vein Occlusion induced Macular Edema	2013	[39]
Tear	Dry eye syndrome	2009	[40]
Tear	Dry eye	2012	[41]
Tear	Dry eye	2013	[42]
Tear	Contact lens dry eye	2009	[43]
Tear	Diabetes and Dry eye	2014	[44]
Tear	Diabetic Retinopathy	2012	[45]
Tear	Dry eye syndrome/Thyroid Associated orbitopathy	2015	[46]
Tear	Primary open angle glaucoma/Pseudoexfoliative glaucoma	2012	[47]
Tear	Alzheimer	2016	[48]
Tear	Multiple Sclerosis	2014	[50]
Tear	Primary open angle glaucoma	2013	[51]
Nipple Aspirate Fluid	Breast cancer	2001	[54]
Nipple Aspirate Fluid	Breast cancer	2004	[55]
Nipple Aspirate Fluid	Breast cancer	2005	[56]
Nipple Aspirate Fluid	Breast cancer	2014	[57]
Breast milk	Diabetes Mellitus	2014	[62]
Cervicovaginal fluid	HIV	2005	[65]
Cervicovaginal fluid	HIV	2014	[66]

Table S1. *Cont.*

Sample	Pathology	Year	Reference
Cervicovaginal fluid	Cervical cancer	2014	[67]
Nasal Secretions	Chronic Rhinosinusitis	2014	[70]
Broncho alveolar lavage	Idiopathic pulmonary fibrosis	2015	[72]
Broncho alveolar lavage	Chronic Obstructive Pulmonary Disease	2013	[73]
Broncho alveolar lavage	Lung Carcinoma	2013	[74]
Broncho alveolar lavage	Acute Respiratory Distress Syndrome	2014	[75]
Broncho alveolar lavage	Lung Adenocarcinoma	2015	[76]
Stools	Non-Alcoholic Fatty Liver Disease	2015	[81]
Stools	Crohn's Disease	2012	[82]

Table S2. Total Identified proteins in each body sample.

Sample Type	Total ID Proteins	Ref. no.
Cerumen	2013	[3]
Saliva	*	[13]
	855	[14]
	180	[15]
	249	[16]
	1243	[17]
	824 **	[18]
	593 **	[19]
	1448	[20]
	148	[21]
	30 approx.	[22]
Vitreous Humor	1111	[23]
	2062	[24]
	252	[25]
	*	[26]
	1242	[27]
	96	[28]
	2482	[29]
	490	[30]
	97	[31]
	412	[32]
Aqueous Humor	154	[33]
	820 **	[34]
	78	[35]
	137	[36]
	*	[37]
	*	[38]
	49	[39]

Table S2. *Cont.*

Sample Type	Total ID Proteins	Ref. no.
Tears	43	[40]
	383 **	[41]
	*	[42]
	616–620	[43]
	357	[44]
	53	[45]
	69	[46]
	*	[47]
	*	[48]
	824	[49]
	185	[50]
	27	[51]
Nipple Aspirate Fluid	64	[52]
	557	[53]
	*	[54]
	41	[55]
	*	[56]
	*	[57]
Breast Milk/Colostrum	*	[58]
	*	[59]
	115	[60]
	82	[61]
	601	[62]
Cervico-vaginal fluid	147	[63]
	15	[64]
	18	[65]
	233	[66]
	712 **	[67]
Nasal Secretions	83	[68]
	111	[69]
	249	[70]
Broncho alveolar Lavage Fluid	376	[71]
	1047	[72]
	423	[73]
	123	[74]
	724	[75]
	1100	[76]
Stools	1534	[77]
	1790	[78]
	1578	[79]
	55	[80]
	*	[81]
	1646	[82]

* Not specified; ** Sum of proteins found in the different experimental groups. They might contain redundant identified proteins.
